# A Standardized Cardiac Protocol for Pediatric Drug Ingestion Hospital Admissions

**DOI:** 10.1097/pq9.0000000000000223

**Published:** 2019-10-23

**Authors:** Erica L. Del Grippo, Shankar Baskar, Seth Gray, Onyekachukwu Osakwe, Adam W. Powell, Jeffrey Anderson, David Spar, Nicolas Madsen

**Affiliations:** From the * Nemours Cardiac Center, AI duPont Hospital for Children, Wilmington, Del.; † Cincinnati Children’s Hospital Medical Center, Cincinnati, Ohio.

## Abstract

To optimize patient resource utilization and safety, we created a standard-of-care guideline for pediatric drug ingestion hospital admissions.

**Methods:**

A multidisciplinary committee developed specific telemetry guidelines for pediatric drug ingestion hospital admissions at a tertiary pediatric hospital. The guidelines stipulated inpatient admission with telemetry monitoring for the following criteria: (1) corrected QT interval (interval between the Q wave and T wave on a standard EKG)≥ 500 ms, (2) ingestion of an antiarrhythmic medication, or (3) ingestion of a tricyclic antidepressant. We created guidelines for electrocardiogram frequency for nontelemetry admissions. We implemented these guidelines in November 2015 in partnership with the Emergency Medicine Department and Poison Control Center. We reviewed medical records of all these admissions between January 1, 2015, and July 31, 2016, and divided patients into preintervention (January 1, 2015 to November 30, 2015) and postintervention (December 1, 2015 to July 31, 2016) groups. We used statistical process control charts and methodology to monitor changes over time.

**Results:**

There were a total of 622 drug ingestion admissions during the study period. We admitted 69 patients (11%) to the cardiac acute care unit (CACU) for telemetry monitoring. The preintervention period included 61 admissions (5.5 CACU admissions per month). The postintervention period included 8 admissions (1.1 CACU admissions per month). This difference reflects an overall absolute decrease of 87%. There was no evidence of an increase in the rate of intensive care unit utilization, rapid response events, or adverse events in the postintervention period.

**Conclusions:**

A standardized admission protocol for pediatric drug ingestions can safely improve resource utilization.

## INTRODUCTION

### Problem Description

Pediatric toxicological ingestions, poisonings, and overdoses are common. Poison control centers report 1.5 million unintentional pediatric ingestions per year.^1^ In the United States, there are approximately 60,000 emergency department (ED) visits per year for unsupervised medication ingestions in children less than 6 years of age.^1^ In addition, there has been a recent increase in opioid-related ingestions in children treated in the ED.^2^

Nationally, hospitals are seeing a rise in poisoning-related hospitalizations.^3^ This observation places an increasing burden on hospital systems to safely care for these patients without overwhelming hospital resources. Given the pharmacodynamics of medications and the concern for cardiac sequelae, admission decisions for patients with drug ingestions can be challenging. As a result, many patients are admitted to an inpatient unit for cardiac telemetry monitoring without specific criteria or justification based on evidence.

### Available Knowledge

Presently, there are minimal pediatric data to determine hospital admission guidelines for drug ingestion. Plumb et al evaluated the efficacy of an observation unit for admission of pediatric patients after ingestion, which demonstrated that poison center recommendations were the basis for primary admission decisions and that 94% of patients with drug ingestion were able to be discharged within 24 hours without a reported adverse event.^4^ Pediatric ingestion patients are screened frequently for QTc (corrected interval between the Q wave and T wave on a standard EKG) abnormalities using electrocardiograms (ECGs). However, screening ECGs are of limited utility and rarely change management in asymptomatic pediatric ingestions.^5^ Adult ingestion patient data suggest that the risk for Torsades de Pointes correlates with QTc of >500 ms.^6^

### Rationale

There are no telemetry guidelines for pediatric drug hospital admissions at this time, and it can be difficult to admit these patients to the proper units. The number of admissions to telemetry is likely secondary to the lack of a protocol and a tendency toward a more conservative approach in the absence of data. Reasons for developing the protocol initially came from the lack of available beds on the cardiac acute care unit (CACU, also known an inpatient ward or “step-down” unit) that were occupied by pediatric drug admissions. The expectation of the success of the protocol came from the lack of historical, anecdotal evidence that these patients had benefitted from their telemetry. No formal studies indicated that these patients required CACU telemetry.

### Specific Aims

**Fig. 1.**
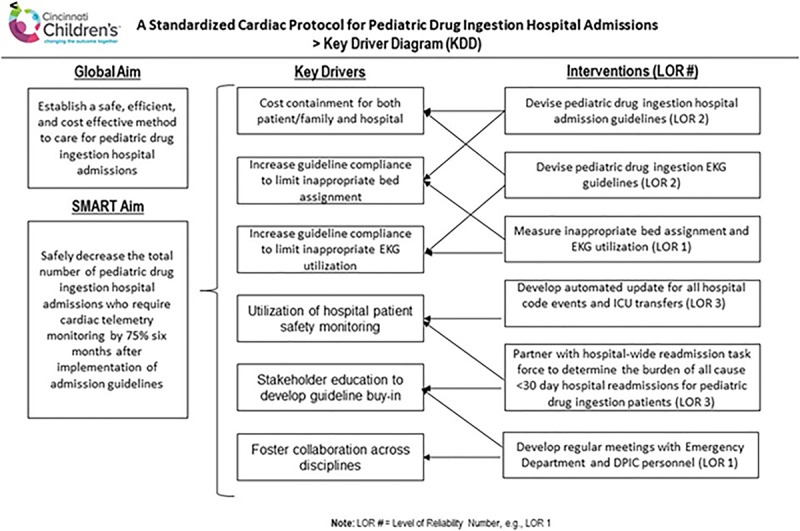
Key driver diagram demonstrates the planning of this project, our primary drivers, and the interventions which led to the attainment of our aims. ICU indicates intensive care unit.

This project aimed to safely reduce the number of pediatric drug ingestion hospital admissions who require inpatient cardiac telemetry monitoring by developing and adopting a standardized protocol to optimize patient resource utilization and safety. Secondary aims included decreased ECG utilization. The smart aim was to safely decrease the number of these pediatric ingestion admissions (PIAs) to the CACU by 75% 6 months after implementation of the new guidelines (Fig. 1).

## METHODS

### Context

This improvement project took place at our hospital and was performed in coordination with the ED and the Drug and Poison Information Center (DPIC). Our facility is a 598-bed hospital that serves a multistate region. The CACU is a 17-bed ward unit and is the only nonintensive care unit in the hospital with the ability to monitor continuous telemetry. A CACU bed has a hospital charge 1.6 times greater than a general pediatrics bed. In our hospital, the ED and the DPIC coordinate drug ingestion triage decisions. Historically, given concerns related to potential cardiac sequelae, we monitored admitted patients on a telemetry bed. These types of admissions are associated with increased cost and resource utilization relative to nontelemetry admissions. This project did not require institutional review board (IRB) approval, given the quality improvement design.

### Patient and Public Involvement Statement

Patients were not involved in the research at any point. The public (including the acute care unit staff, DPIC, and ED staff) was made aware of the initial presentation of the guidelines. ELD, SB, SG, OO, AWP, DS, and NM developed the research question. Given the background of the physicians as cardiac fellows, electrophysiologist, and acute care pediatric cardiologist, this allowed us to discuss a meaningful design for the acute care unit with telemetry involvement. The acute care unit staff, DPIC, and the ED staff were emailed about the guidelines and given direct information via telephone on the design of the study. Patients were not involved in study recruitment. The public was not asked to assess the burden of the intervention and time required to participate in the research.

### Intervention

We obtained baseline data by review of all admitted pediatric patients (less than 18 years old at hospital admission) with the diagnosis of drug ingestion over 11 months (January 1, 2015 to November 30, 2015). A multidisciplinary team (representation from cardiology, emergency medicine, and poison control center) evaluated the types of patients who were admitted to the CACU for telemetry, and those admitted to a nontelemetry general ward unit or an intensive care unit. We excluded all patients who ingested household cleaners and those with smoke inhalation as their primary diagnosis.

This multidisciplinary team then created specific cardiac telemetry guidelines for PIAs. The guidelines stipulated admission to the CACU for telemetry monitoring only if any 1 of the following criteria were met: (1) QTc ≥ 500 ms, (2) ingestion of an antiarrhythmic medication, or (3) ingestion of tricyclic antidepressants. These guidelines were reviewed and approved by 2 heart institute electrophysiologists (authors JA and DS). We created guidelines for ECG frequency for nontelemetry admissions and stipulated: (1) patients with an initial QTc > 440 ms should obtain an ECG every 8 hours until the QTc normalized and (2) patients with an initial normal ECG should not receive additional scheduled ECGs. Furthermore, if the QTc remained abnormal greater than 24 hours, a cardiology consult should be requested. These guidelines were discussed and shared with DPIC and ED faculty and staff, and the CACU staff (nursing, nurse practitioner, fellows, and faculty). There were no additional changes to the protocol during the intervention phase.

**Fig. 2.**
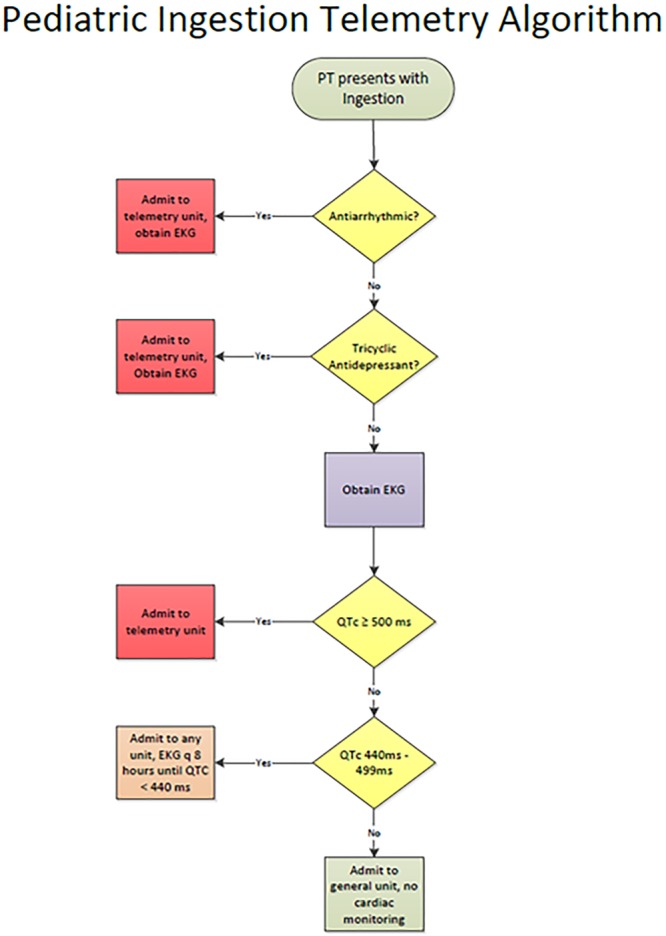
The algorithm demonstrates the protocol used for the education of our staff and the management of patients with PIAs. PT, patient.

Before the implementation of these guidelines, the improvement process included unit and stakeholder education. Critical to this education was the inclusion of the cardiology and ED charge nurses. We educated the staff on the protocol and the type of patient who required telemetry via a management algorithm (Fig. 2). We held multiple cycles of education, given the multitude of staff involved. Separately, we created specific education for the staff of the DPIC. Given the historical role of the DPIC in advising on disposition and need for monitoring, this was an important step to set expectations. Also, the process involved education for the general pediatric hospitalist teams who now would be caring for a larger proportion of the patients admitted for ingestion, including those who had historically been on the CACU. This transition was viewed favorably by the pediatric hospitalist team as they understood the rationale, appreciated the clear escalation and ECG guidelines, and felt supported should they require subspecialty consultation.

### Counterbalance Measures

Once we created and distributed the new guidelines, we studied the improvement over 6 months (January 1, 2016 to July 31, 2016). We identified counterbalance measures including postintervention adverse events, pediatric intensive care unit (PICU) admissions, and transfers, length of stay, number of EKGs, and number of less than 30-day readmissions. DPIC provided a database of hospital admissions secondary to ingestion. We reviewed the completeness and accuracy of all patients by matching admission date and medical record number. We divided the patients into 2 groups: preintervention (January 1, 2015 to November 30, 2015) and the postintervention (January 1, 2016 to July 31, 2016) based on their admission date. We reviewed the QTc on the initial ECG, number of ECGs recorded, and drugs ingested.

In addition, the following authors, ELD, SB, SG, AWP, OO, and NM reviewed all patients. We collected patient admission location during the chart review. Those admitted to the PICU were reviewed to determine the reason for admission. If patients were admitted to an inpatient nontelemetry ward, and then subsequently transferred to the PICU, we also collected the reason for the transfer. We screened patients for adverse events, including ventricular arrhythmia or death.

### Analysis

We identified patients who met our inclusion criteria for the study and divided them into pre- and postintervention groups as noted above. The operational definition of the numerator was patients admitted to the CACU for pediatric drug ingestion, and the operational definition of the denominator was all PIAs no matter the location for each study month. Results were displayed using a P chart (statistical process control chart). In addition, we assessed the patients’ “appropriateness” for the CACU according to our guidelines.

### Ethical Considerations

There were no ethical objections to this improvement proposal. However, constant reevaluation of the protocol was performed to determine whether any patients experienced ventricular arrhythmias, code event, or mortality when admitted to a nontelemetry unit.

## RESULTS

**Fig. 3.**
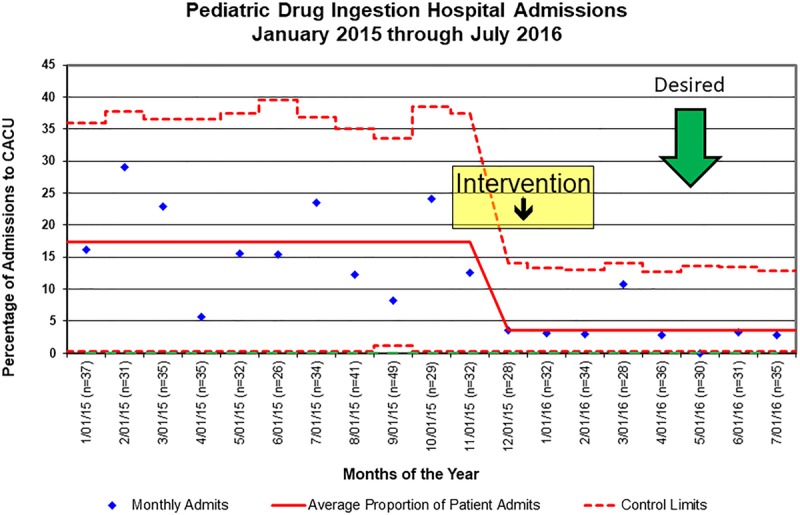
This P chart demonstrates the percentage of drug ingestion admissions to our acute care unit over the study period. There is a decrease in admissions to this unit after the intervention as noted by the chart.

There were a total of 622 PIAs to our facility during the study period, of which we admitted 69 patients (11%) to the CACU for telemetry monitoring. The preintervention period included 61 of these admissions (88%, and 10% of total). This population represented 5.5 CACU admissions per month. The postintervention period included 8 total CACU admissions (12%, and 1% of total) and represented 1.1 CACU admissions per month. This difference between the pre- and postintervention period reflected an overall absolute decrease of 87% (Fig. 3).

Among the overall total ingestion admissions, there were 115 admissions to the PICU during the study period (19%). The preintervention period included 84 of these admissions (73%), which represented 7.6 PICU admissions per month. The postintervention period included 31 admissions (27%), which represented 4.4 PICU admissions per month. During the preintervention period, the most common reason for PICU admission was for altered mental status (42 patients), followed by “need for telemetry” (13 patients). There were a total of 2 PICU transfers from the hospital ward during this period for possible seizure and agitation. In the postintervention period, the most common reason for PICU admission was for altered mental status, followed by hypotension, respiratory compromise, and seizure. There were 3 PICU transfers from the hospital ward during this phase. All 3 of these transfers were unrelated to telemetry monitoring and were secondary to altered mental status and behavioral issues. There were no adverse events in the postintervention period. The median length of stay for all ingestion hospital admissions in the preintervention period was 2.33 days per patients. The median length of stay remained stable at 2.27 days per patient in the postguideline period. There were no less than 30-day hospital readmissions during the entire study period. Regarding ECGs completed, the rate remained unchanged over the study period with 1.3 EKGs per admission in the preintervention period and 1.5 EKGs per admission in the postintervention period.

In addition, we reviewed inappropriate CACU ingestion admissions per study guidelines. Of the total 69 CACU admissions, 62 patients (90%) could be determined to be inappropriate. The preintervention period included 59 of these inappropriate admissions (95%, 5.4 inappropriate admissions per month). The postintervention period included 3 such admissions (5%, 0.4 inappropriate admissions per month). Utilizing the study guidelines retrospectively, the median percentage of ingestion admissions to an inappropriate unit decreased from 15% to 5%.

## DISCUSSIONS

### Summary

Our quality improvement collaborative project demonstrated that routine cardiac telemetry monitoring of pediatric drug ingestion hospital admissions to a tertiary pediatric hospital may only be required in a tailored subset of these admissions and the remaining may be safely excluded from this monitoring requirement. Specifically, unless patients meet predetermined, and intentionally narrow, criteria, as highlighted by our guidelines, cardiac-specific surveillance is not required. Our project demonstrated that these admissions could be safely cared for on the general hospital ward without an increase in adverse events, resources allocated, or increased hospital length of stay.

PIAs are increasing in frequency and are at risk to further escalate in the near term given the rising opioid epidemic. Evidence demonstrates that more adults are prescribed medications, such as opioids, which place the pediatric population at increased risk of accidental and intentional ingestion.^1,2^ Specifically, opioid exposures are common in children less than 5 years old.^3^

The cost associated with PIAs is an additional concern. In a study by Levine et al, the median charge per patient for unintentional ingestion of ADHD medications was between $4,500 and $6,000. They estimated that the annual charges across the United States yearly for these admissions would be $24 million.^7^ By decreasing the number of patients admitted to cardiac telemetry units, there are potential cost savings to the patient and family, and the hospital. At our hospital, we estimate that the daily cost of these admissions decreased by 17% per month after guideline initiation. Furthermore, we estimate that patient charges at our hospital would decrease by 40% per day for the approximately 4.4 families per month who could be admitted to the general pediatric ward instead of the CACU.

Currently, there are minimal pediatric data for drug ingestions telemetry guidelines. The only available literature involves adult medicine, which demonstrates that the role of telemetry in guiding patient management is overestimated.^8^

### Interpretation

Our protocol was created by expert consensus and spread via key stakeholder education. The members of the care team most crucial to the success of the guidelines were the charge nurses in the ED and the CACU. Also, a partnership with the cardiology fellows, who helped guide the appropriate disposition of each patient, was necessary. The significant decrease in CACU admissions in the postintervention phase reflects the successful implementation. The algorithm initiation was simple and effective, and the product of a multidisciplinary cohort of stakeholders.

Regarding the usability of the guidelines, if we applied the guidelines retrospectively to patients during the preintervention phase, there were 59 inappropriate admissions to the CACU during the 11 months. After the implementation of the protocol, there were only 3 inappropriate admissions over 6 months. This result was less than 2% of all admissions for drug ingestion to the hospital. We believe this highlights the reliability and simplicity of our approach.

Although the study period ended in July 2016, the medical leadership of the CACU (author NM) has not observed a change in the admission practices for pediatric drug ingestion. These patients are only very rarely admitted for telemetry, less than 1–2 per month, and only by the guidelines put in place by this quality improvement project.

### Strengths

We conducted this study at a large pediatric center with 622 patients. This study discusses 1 of the current problems in pediatric medicine; unintentional drug ingestions. It shows multidisciplinary teamwork.

### Limitations

Limitations of this study include both compliance with the protocol and change in practice. There was not 100% compliance upon review, and it is unclear why those 3 patients were not admitted according to the guidelines. Although there was an initial concern that an increased number of patients would be admitted to the PICU postintervention, given it is the only other place in the hospital with telemetry capacity, this did not occur. Specifically, the rate of PICU admission for drug ingestion did not increase during the intervention phase.

Furthermore, we also did not measure an increase transfers from the hospital ward to the PICU during the postintervention phase. Also, because of the variability of charge data and lack of accuracy, we did not include these measures in our results. We did not believe it was in the scope of our work at this time. In addition, we did not explore or separate intentional versus accidental ingestions when reviewing our data. Lastly, although these guidelines were successful at our institution, it is not known how generalizable the results might be to other pediatric hospital centers.

## CONCLUSIONS

A pediatric hospital admission drug ingestion protocol can safely decrease the frequency of admissions that require cardiac telemetry monitoring. As a result, there is an opportunity to decrease costs and resource utilization associated with care for this increasingly common patient admission type. To our knowledge, our improvement project is the first to investigate the most effective, efficient, and safe approach to caring for pediatric drug ingestion hospital admissions.

## ACKNOWLEDGMENT

The datasets generated during and/or analyzed during the current study are available from the corresponding author on reasonable request.

## DISCLOSURE

The authors have no financial interest to declare in relation to the content of this article.
